# Rationing of nursing care: A concept analysis

**DOI:** 10.1016/j.heliyon.2023.e15861

**Published:** 2023-04-28

**Authors:** Tayebeh Moradi, Mohsen Adib-Hajbaghery, Mansour Dianati, Fatemeh Moradi

**Affiliations:** a Trauma Nursing Research Center, Kashan University of Medical Sciences, Kashan, Iran; b Department of English Language and Literature, Allameh Tabataba'i University, Tehran, Iran

**Keywords:** Rationing of nursing care, Concept analysis, Nursing

## Abstract

Rationing of nursing care (RONC) refers to necessary nursing tasks that nurses refuse or fail to do because of limited time, staffing level, or skill mix. As an important process factor, it affects the quality of patient care. The concept of rationing of nursing care has not yet been clearly defined and analyzed and there are different views regarding this issue. Using Walker and Avant's eight-step method, this concept analysis was conducted to analyze the meaning, attributes, dimensions, antecedents, and consequences of nursing care rationing. The literature was collected by searching in electronic databases including PubMed, ScienceDirect, Web of Science, Scopus, and Google Scholar with no date limitation. Both qualitative and quantitative studies on rationing of nursing care, which were open-access and published in English, were included in this study. Thirty-three articles were investigated in the present study. The four defining attributes of RONC included the duty of performing nursing care, dealing with problems of doing nursing care, decision-making and prioritizing, and outcome. The antecedents included nurse-related, organization-related, care-related, and patient-related antecedents. A theoretical definition and a conceptual model of RONC were developed. The attributes, antecedents, and consequences of RONC identified in this study can be used in nursing education, research, and managerial and organizational planning.

## 1 Introduction

In recent decades, the rate of health expenditure growth has exceeded the rate of economic growth. Policymakers and healthcare managers have been compelled to use strategies such as prioritizing and rationing, and invested in more cost-effective, affordable, patient-centered, and safe health services. The World Health Organization (WHO) has also emphasized the importance of rationing as a precondition for global health coverage [1]. Referring to the vastness of the health needs of communities and limited resources, some experts consider health service rationing inevitable [2].

Rationing of nursing care was first introduced by Schubert in 2007. She defined rationing of nursing care as “withholding of or failure to carry out necessary nursing interventions for patients due to a lack of nursing resources such as staff, skill, and time” [3].

The nature of nursing care rationing is different from the general definitions of rationing and health services rationing, and nursing articles have offered a different definition of this concept. In some articles, it is the same as missed nursing care (delay in or omission of nursing care activities) [4]. However, the concept is defined in other articles as the process of decision-making of nurses in the context of the nursing care prioritization [5]. The definition of rationing provided by Schubert does not seem to be complete either, as she conceptualizes it as a negative concept, which encompasses withholding and not performing some beneficial or required interventions. Furthermore, the consequences of nursing care rationing are not widely discussed, and the definition presented by Schubert addresses only the inadequacy of manpower, skills, and time as the essential resources for nursing care [3]. Nonetheless, several additional factors may affect nursing care, and its rationing might bring further consequences [[6], [7], [8], [9]]. Given the fact that the concept of nursing care rationing is not clearly explained, the present study aimed to clarify the concept, its attributes, antecedents, and consequences.

## 2 Methods

### 2.1 Study design

This concept analysis was conducted using Walker and Avant's eight-step method to analyze the meaning, attributes, dimensions, antecedents, and consequences of nursing care rationing. Concept analysis, while distinguishing a concept from other similar concepts, develops it as fully as possible [10]. This approach aims to select the concept, determine the purposes of analysis (which was discussed in the introduction), identify all uses of the concept, specify the defining attributes of the concept, construct a model case, create a borderline, contrary, and invented case, identify the antecedents and consequences of the concept, and define the empirical referents of the concept [10].

### 2.2 Data collection

An extensive online search and literature review was conducted to find relevant data on rationing of nursing care. Several online databases including PubMed, ScienceDirect, Web of Science, Scopus, and Google Scholar were scrutinized. Keywords such as “rationing”, “ration”, “nursing”, and “nurse” were searched in the above-mention databases. These keywords were searched in the title, abstract, and keywords section of the studies. The search protocol was not limited to any date, and the Boolean operator “AND” was used to combine the keywords. Both quantitative and qualitative studies, which had investigated rationing of nursing care and were published before 2021 in English and their full-texts were available, were included in the study. All irrelevant and duplicated studies were excluded. After excluding duplicates, 33 related articles were reviewed to extract the definitions, attributes, antecedents, and consequences of the intended concept.

## 3 Results

### 3.1 Definition of the concept and identification of its uses

Being derived from the Latin root “ration”, the word “rationing” cannot be defined comprehensively and sufficiently. Longman dictionary defines “ration” —as a noun— as “a fixed amount of something that people are legally allowed to have in case of shortage, for example, during a war” or “an amount of something that you think is reasonable or normal”. As a verb, it is also defined as “to control the supply of something because there is not enough” and “to allow someone to have only a small amount of something (like food or gasoline) or less than they would like because there is not enough” [11]. The Oxford Learner's Dictionary also defines rationing as “the policy of limiting the amount of food, fuel, etc., that people are allowed to have when there is not enough for everyone to have as much as they want” [12].

Rationing systems were first for food and then extended to energy sources such as oil, gas, water, and electricity [13]. According to Scott et al. rationing means restricting the distribution of scarce resources or allocating a certain amount of resources to individuals, indicating that the allocated resources are scarce and, thus, will not be sufficient to provide whatever is needed. Therefore, the distribution of such insufficient resources should be controlled by rationing [14].

Health services rationing was primarily described in the medical field and was used to refer to resource allocation [15]. The online Medical Dictionary defines health services rationing as the limitation of access to or the equitable distribution of medical services and available healthcare resources through various gatekeeper controls and planning [16]. Rationing might explicitly or implicitly restrict people's access to some useful or potentially useful health services because of budget constraints [1]. Explicit rationing is implemented by officials or institutions based on very well-defined policies, protocols, and conscious decisions for withdrawing some beneficial caring measures or medical treatments of certain persons [14], and is a form of resource allocation. An example is a system designed for the distribution of available transplantable organs [17]. Decisions are explicit, are usually made at the macro level, and have an administrative and political nature [15]. Implicit rationing is, however, implemented indirectly by individuals and through their practice and is not based on normative institutional or professional principles [14]. Implicit rationing immediately affects the patients and is usually influenced by the healthcare providers' personal experiences and their considerations of specific situations [15]. Implicit rationing occurs in clinical settings and is based on the context within which the healthcare professionals work and are influenced by budgetary constraints or even unrecognized individual biases. An example is a physician who must decide which patient will fill the last intensive care unit (ICU) bed [17].

Health services rationing might be considered negative if it withholds a beneficial service or treatment from a patient or a group of patients. For example, patients might be deprived of receiving a beneficial treatment if the hospital's committee on pharmaceuticals decides to restrict the use of an effective but expensive medication to patients with certain criteria [17,18]. However, rationing might be regarded in a less negative way when it restricts access to something or services with potential but not proven effects, especially when the costs far outweigh the potential benefits. Rationing can be seen in this way if it is implemented based on clear rules and principles [1,14,19,20].

Schubert et al. (2005) first introduced the concept of nursing care rationing in a study that investigated the concept in Switzerland and aimed to map the level of care provided in Swiss acute care centers. They also clarified the concept of rationing and defined it as “the withholding of or failure to carry out necessary nursing measures for patients due to a lack of nursing resources (staffing, skill mix, time)” [3,21]. Rationing of nursing care is also defined as the suspension or discontinuation of some aspects of care when resources are limited and tasks are assigned incorrectly [22]. According to the conceptual framework developed by Schubert, rationing of nursing care can include monitoring therapeutic, preventive, rehabilitative, and supportive measures [6].

Rationing of nursing care does not explicitly exclude patients from receiving specific healthcare services. Conversely, despite having a limited number of staff or too many demands by the institution, the nurses are still expected to provide the full range of nursing care activities. Unlike physicians, nurses are not explicitly asked to apply rationing or specific cost-effectiveness considerations, and still are expected to meet patients’ needs fully [14]. Therefore, rationing of nursing care is usually implemented implicitly [21], without any clear rules and regulations for guiding the rationing processes. In fact, the individual nurse decides how limited resources (such as time and skills) are distributed among the patients and determines who should receive a specific necessary nursing care [7].

Nurses need sufficient time, staffing level, and skill mix to provide quality nursing care [8]. Therefore, they may refrain from doing necessary nursing cares for all patients when they are not able to allocate a certain amount of time and energy to each patient and, hence, rationing of nursing care will occur [[23], [24], [25]]. Thus, rationing of nursing care is a process in which nurses prioritize the needs of patients in situations where there are numerous needs and insufficient resources. Then, nurses decide not to complete all patient care activities, delay some aspects of care or leave out some others [8,9,26,27]. In other words, rationing of nursing care can be defined as a planned decision-making process for providing incomplete care. This decision is made before caring process and is according to nurse-, patient-, organization-, or care-related conditions. Rationing of nursing care, thus, would lead to missed, unfinished, and delayed nursing care which, as malpractice, can affect patient outcomes negatively [3,8,13].

### 3.2 Relevant concepts

Rationing and resource allocation are related but distinct concepts. In both cases, specific criteria are applied to how available resources are distributed to optimize outcomes or meet procedural justice requirements. From an ethical point of view, however, resource allocation is a concept that is neutral regarding its moral consequences, and refers to any approach for the distribution of either abundant or scarce resources. On the contrary, rationing results from resource scarcity and leads to less than optimal benefits for some people [14]. In fact, resource allocation is more related to the allocation of healthcare providers such as the number of nurses available in the institution (i.e. nurse-patient ratio), whereas rationing of nursing care refers to the individual nurse's decision on how much care s/he would provide for a patient or a number of patients [14].

Prioritization of care is another concept that, while closely related to both nursing care rationing and resource allocation, is different from both concepts. Prioritization of care helps nurses organize their work and maximize patient outcomes. Prioritization of care is the decision made by nurses during their interaction with patients to address which caring needs are among many potentially competing needs and options. Therefore, the concept does not have a negative meaning [28].

Missed care, unfinished nursing care, and delayed nursing care are some additional terms that are sometimes used as synonyms of nursing care rationing. However, they are different from rationing or might be considered as the consequences of nursing care rationing [9,13]. The term rationing care is related to, but distinct from, missed care, unfinished nursing care, and delayed nursing care. Rationing involves a planned and reasoned decision to deny access to care. In nursing, the notion of insufficient resources is addressed as a reason of rationing in this definition. However, other concepts may not be planned or reasoned and are not caused by resources [[28], [29], [30]]. These concepts are frequently replaced but rationed care is the process of decision-making of nurses in the context of nursing care prioritization which results in an outcome such as unfinished or delayed nursing care [5,28].

### 3.3 Definition of the attributes of nursing care rationing

The core of concept analysis is to determine the defining attributes of a concept. A concept usually has several defining attributes, the most appropriate of which must be determined in concept analysis [10]. These attributes differentiate the intended concept from similar or related concepts. The most important attributes of nursing care rationing include the duty of performing nursing care [8]; dealing with the problems of doing nursing care [14]; clinical judgment, decision-making and prioritization; and the outcome which usually is the restricted access of patients to complete care [8,9,27].

*1-The duty of performing nursing care:* Nurses have a key role as healthcare providers and act as the front line of the healthcare system. They play the roles of planners, coordinators, providers, and evaluators of nursing care. They carry out nursing care as well as a myriad of interventions prescribed by other providers to treat illnesses and complications, and promote patient health and well-being. If the flow of care provided by nurses is blocked, patients may receive incomplete or inappropriate care [13].

*2-Dealing with the problems of doing nursing care:* Nurses are often forced to provide care in the face of limitations and inadequate resources of nursing or the organization. In such conditions, they may have to distribute their time and energy fairly among the patients [8,9,26,27].

*3-Clinical judgment, decision-making and prioritization*: Decision-making is a contextual, continuous, and evolving process where data are collected, interpreted, and evaluated to ultimately select an evidence-based action [30]. Nurses often make decisions based on their assessment of the patient's conditions and needs and, depending on the circumstances, they judge, prioritize, and plan for the provision of nursing care [31]. Based on the contextual variables, antecedents, and possible outcomes, nurses choose which of the patient's competitive needs should be addressed primarily, and how to allocate their time to patients to obtain the best possible outcomes [31].

*4-Outcome:* When the required resources are insufficient, nurses turn to nursing care rationing and decide how to distribute available resources (most importantly their time, energy, and skill mix) among patients. Rationing usually leads to the missed or delayed care or may restrict the access of patients to complete care [7,25].

### 3.4 Construction of a model case

The model case is an example of using the concept that demonstrates all its defining attributes, and contributes to the better articulation of the concept's meaning. The model case helps readers understand how to interpret and define a concept [10].

Ms. Akbari is a nurse in the gastrointestinal surgery department. She is assigned the responsibility of caring for five patients (the duty of performing nursing care). One patient's dressing should be changed at the beginning of the shift. Another patient also underwent surgery yesterday and will be discharged today at 10 o'clock after a medical visit and dressing change. Three other patients must be prepared to go to the operating room by 10 a.m. Depending on the circumstances, the large number of care measures that need to be done, and the lack of time (dealing with problems), the nurse decides (clinical judgment and decision-making and prioritization) to first prepare the three patients who must go to the operating room. It is about 10 a.m. when the doctor comes to visit the first patient and discharges her. At 10:30 a.m., the nurse is preparing to change the first patient's dressing and provide discharge training when she is informed that the three patients must immediately be admitted to the ward, and one of the new patients is assigned to her. Suddenly, another nurse runs up to her and asks her help to manage a patient who has abruptly got unconscious. Because of the situation, the nurse decides to postpone changing her patient's dressing to the end of the shift (delayed care), and removes the discharged patient's education from her schedule (missed care). In this way, the access of some patients to nursing care is restricted. At 12 o'clock, the three patients who have undergone surgery are returned to the ward, and because the nurse does not have enough time to provide them with full care, she decides to provide each patient only with some basic cares (incomplete care).

### 3.5 Construction of a borderline case

Borderline cases refer to those cases that contain most but not all defining attributes of the concept [10]. Mr. Ahmadi is a nurse in the emergency department. The nursing care of three patients has been assigned to him (the duty of performing nursing care). The first patient is on complete bed rest and his position should be changed every 2 h. The second patient suffers from gastrointestinal bleeding and the third one has a heart attack. Another nurse asks him to change her patient's dressing every 2 h (no duty to carry out nursing care).

He does the nursing care of his three patients. Given the heavy workload and lack of time (dealing with the problems), the nurse decides (decision-making) to do the dressing of his colleague's patient every 3 h (delayed and incomplete care).

### 3.6 Construction of a contrary case

The contrary case does not have any of the defining attributes of the concept and is an example of what the concept lacks [10]. Mr. Osuli is an orthopedic nurse and three patients are assigned to him today (the duty of performing nursing care). His first patient is a trauma patient who is now in stable condition and has been prepared to be transferred to the operating room for femur surgery. The second patient has also been admitted with a lower limb fracture. He is in a stable condition and is in traction now. The third patient is admitted for removing an internal fixator that was implanted in his left arm a year ago. Her fixator was removed the previous day and she will be released today after changing her dressing.

At the beginning of the shift, Mr. Osuli examines all three patients and concludes that they are all in good condition and that he can provide all the necessary nursing care for each of them (having no problem in carrying out nursing care). After greeting and primary assessment, he decides to start with the patient who is going to the operating room (clinical judgment and decision-making). He checks the patient's preparations, talks with him about the surgery and aftercare, and also gives him a pamphlet about postsurgical care. Mr. Osuli then talks to the second patient for a while, examines the proper functioning of his traction, answers his questions, and teaches him how long the traction would last, as well as about the importance of proper alignment of the fractured limb and the significance of moving other parts of the body while being in bed. After escorting the first patient to the operating room, Mr. Osuli changes the third patient's hand dressing, examines his wound healing, teaches him about wound care, infection prevention, and hygiene at home, and prepares him for discharge. Therefore, the nurse does not impose any rationing or restrictions on nursing care.

### 3.7 Identification of antecedents and consequences of the concept

#### 3.7.1 Antecedents of nursing care rationing

Antecedents are conditions and events that exist or happen before the intended concept [10]. Antecedents of nursing care rationing can be classified into four categories of nurse-related, organization-related, care-related, and patient-related antecedents.

*Nurse-related antecedents* are inadequate physical and mental health, fatigue, headache, depression [32], dissatisfaction with life [33], dissatisfaction with one's current position in the workplace [34], burnout and low job satisfaction [35], poor interpersonal relationships and communication [36], low controlled performance [37], low emotional intelligence, and low empathy. Low emotional intelligence and empathy also negatively affect the relationships of nurses [37] as well as their experience, education, skills, autonomy, and responsibility [3].

*Organization-related antecedents* refer to inadequate and inexperienced workforce [32,38], low nurse-patient ratio [30], high workload caused by the high number of patients per shift and the number and complexities of provided nursing cares [21,27,36], lack of resources needed for patient care [3,38], ineffective delegation of specific tasks [34], time-consuming documentation tasks and old information technologies [36], inadequate supervision [38], weak and inappropriate organizational support such as inadequate in-service educations and not meeting the needs of patients and nurses [36,37], poor teamwork [3,36,39], lack of proper doctor-nurse communication [36], unfair treatment by nurse managers [34], legal and organizational restrictions [40], presenteeism or working during one's sickness [41], low autonomy and control over practice [3,37], lack of a professional work setting [9,24,37], inadequate and unsuitable materials and equipment [36], workplace culture, emphasis on specific tasks by the department manager [3,26], local and national guideline, and procedure [3].

*Care-related antecedents* include nursing cares which take more time [30,42], care for psychosocial activities such as providing patient education, emotional support, and communication with the patients [[13], [14], [15]], activities related to patients’ mobility and hygiene (such as oral hygiene and bathing) [9,21], and activities with no immediate effect on the patient [9,26]. Most nurses often meet patients' basic medical needs, but usually ration the needs that do not have an immediate impact on patients. For example, they usually provide medication to patients but ignore their education [15,26].

*Patient-related antecedents* include type and severity of illness, number of health problems, the acuteness of patient condition, comorbidity and risk factors [3], patients' lack of cooperation, non-compliance, and aggressive behaviors. Furthermore, substance abuse, the very low or high age of patients, and the culture and ethnicity of patients [3] can affect the behavior and performance of the nurses in providing care [6,25].

#### 3.7.2 Consequences of nursing care rationing

Consequences are conditions or events that occur after the presence of the concept [10]. The consequences of nursing care rationing can be divided into three sub-categories of nurse-related, organization-related, and patient-related consequences.

*Nurse-related and organization-related consequences* of nursing care rationing include low job satisfaction [9], role conflict [24], feelings of guilt, worry, and moral distress [24,26,43], intention to leave the job, and high turnover of nurses [35].

*Patient-related and care-related consequences* of nursing care rationing include decreased quality of care [25,30,34], decreased patient satisfaction [6,21,24], and negative patient outcomes (decreased patient safety, medication errors, falls, nosocomial infections, accidents, pressure ulcers, and death) [15,21,25,44].

### 3.8 Definition of empirical referents

Concepts and their attributes are abstract and, therefore, cannot be easily measured without reliable empirical indicators. According to Walker and Avant, empirical referents are “classes of actual phenomena that by their existence or presence demonstrate the occurrence of the concept itself.” Although empirical referents are not necessarily the tools for measuring a concept, they are the means by which one can recognize or measure the defining characteristics of a concept and help instrument development and research on the concept [10]. A number of instruments are developed to measure nursing care rationing and some parts of the nursing activities that are eliminated after nursing care rationing [15]. Basel Extent of Rationing of Nursing Care (BERNCA) is a special measurement tool that has been developed in Switzerland to measure the extent to which the tasks are withheld through rationing [3]. The BERNCA includes 20 items that mostly refer to those nursing cares, which are often omitted or ignored. This scale has five subscales (i.e. activities of daily living (ADL), caring support, rehabilitation/instruction/education, monitoring-safety, and documentation) and has been tested especially in acute care settings [3]. The BERNCA was later revised and published as BERNCA-R [21]. It has also been adopted and called BERNCA-NH to measure the extent of implicit rationing of nursing care in nursing homes [45,46]. The BERNCA has been translated and used in several languages [9,40], and Perceived Implicit Rationing of Nursing Care (PIRNCA) is also a revised version of BERNCA [47]. This 31-item version consists of special nursing care activities needed to be performed by RNs who provide nursing care for adult patients, and is used frequently [15,47,48]. However, these instruments measure only some aspects of nursing care and have not been used widely [15].

### 3.9 Definition of the concept

**Fig. 1:**
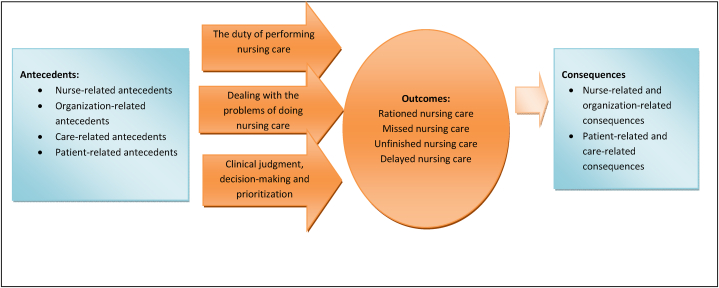
Nursing care rationing.

Based on the present analysis, rationing of nursing can be defined as the process of nurse planned decision-making and prioritization of incomplete care (or limited access to care) among patients according to nurse-, patient-, organization-, or care-related conditions that can lead to missed, unfinished, and delayed nursing care. All of these have negative effects on the outcomes of patients and are considered malpractice. Fig. 1 shows the conceptual model of rationing of nursing care developed based on the findings of this study (insert Fig. 1).

## 4 Discussion

In this study, Walker and Avant's method was applied to analyze the concept of nursing care rationing. As found out in the study, the concept of nursing care rationing is different from the concept of rationing in general or even from health service rationing. Rationing refers to the restriction of the distribution of scarce resources and implies that the resources determined to be allocated are scarce and, thus, cannot be provided adequately [14]. Health service rationing is a mechanism used to allocate healthcare resources. Because of limited budget, it restricts the access of some people to useful or potentially useful health services [1]. However, nursing care rationing totally differs from rationing as a general concept.

According to the analysis, nursing care rationing can be defined as an intentional decision for the shortcomings of nurses in doing their formal duties assigned to them for the patients. Although these intentional shortcomings are decided by the individual nurses, the biased or somewhat unethical decision of them to restrict or withhold some necessary and beneficial nursing care from a patient or a group of patients [8] is in fact their reaction to the high workload and problems imposed on them by the organization.

Based on the analysis, rationing of nursing care was revealed to be a process involving antecedents and consequences. Several nurse-, organization-, care-, and patient-related factors might act as the antecedents of nursing care rationing [24,25]. All these factors will eventually lead to a lack of essential nursing resources such as staff, time, and skill mix and, consequently, force a nurse to prioritize one nursing care/patient over another based on an individual's judgment. Then, the nurse attempts to distribute their limited energy and time according to such priorities [[6], [7], [8]]. Nurses' fatigue, dissatisfaction with their current position in the workplace [34], poor interpersonal relationships and communication [35], inadequate and inexperienced workforce [32,37], high workload, inadequate supervision [37], weak organizational support, care needing psychosocial activities such as providing patient education, activities related to the patient's mobility and hygiene, activities with no immediate effect on the patient [9,26], and the patient's lack of cooperation are among the most important antecedents of nursing care rationing. Nursing care rationing also will lead to consequences for the nurses, organizations, and patients. The job dissatisfaction of the nurses [9], role conflict [24], moral distress [24,26,43], intention to leave [35], and the patient's dissatisfaction with the provided care are among the most important consequences of nursing care rationing.

## 5 Study limitations

Although the researchers tried to include all relevant articles in the review, many articles could not be included that was due to the researchers’ lack of access to some databases and, thus, only open-access English articles were examined. Based on the knowledge of the researchers, there is not an adequate definition of rationing of nursing care, even after searching different databases and sources. Furthermore, it is recommended that researchers conduct more qualitative studies to develop an appropriate definition of nursing care rationing.

## 6 Conclusion

The concept of nursing care rationing is different from the concept of rationing in general or even from health service rationing. According to this concept analysis, nursing care rationing is an intentional decision made by a nurse to limit or withhold necessary nursing care from a patient or a group of patients. This biased and unethical decision is made by the nurses in response to the high workload and problems the organization imposed on them. Several nurse-, organization-, care-, and patient-related factors might act as the antecedents of nursing care rationing. Not only does nursing care rationing affect nursing care negatively, but it also decreases patient safety and results in unsuitable consequences for the nurse and the organization. Nurse Managers are recommended to identify the antecedents and contextual variables leading to nursing care rationing and prevent nursing care rationing by improving the nurse-patient ratio and decreasing the workload nurses. Nurse educators are also suggested to emphasize patient rights and ethics of care in educating the nurses and nursing students and ask them not ration nursing care in response to the working conditions.

## Author contribution statement

All authors listed have significantly contributed to the development and the writing of this article.

## Data availability statement

No data was used for the research described in the article.

## Declaration of competing interest

The authors declare that they have no known competing financial interests or personal relationships that could have appeared to influence the work reported in this paper.
